# Journalistic Laundry Work

**Published:** 1914-09

**Authors:** 


### Journalistic Laundry Work.

In no censorious spirit but because they realize that a medical journal of general scope should publish medical news as well as medical science, several readers have asked why we have made no comment on various scandals and tragedies involving the medical profession and given wide publicity in the lay press. The reason for our silence is an objection to washing dirty linen or even to hanging it on our clothes line after it has been washed and every disagreeable detail wrung out of it by periodicals of different kind. There are many cases in which unpleasant subjects must be discussed in solving vital problems of professional ethics and policy. Such duties, we do not intend to shirk. The cases to which we allude are those in which physicians are brought into notoriety by their own passions or those of others and in which the moral to be drawn is obvious or else, so dependent upon facts which we have no personal means of verifying, that it seems unwise to assume either or any side of a controversy and to moralize upon it. We could very easily make the tint of our cover penetrate the whole of the journal. It would be profitable to do so, as has been established by actual experience in medical journalism. But, all things considered, we believe our readers will agree that a conservative policy is better.

